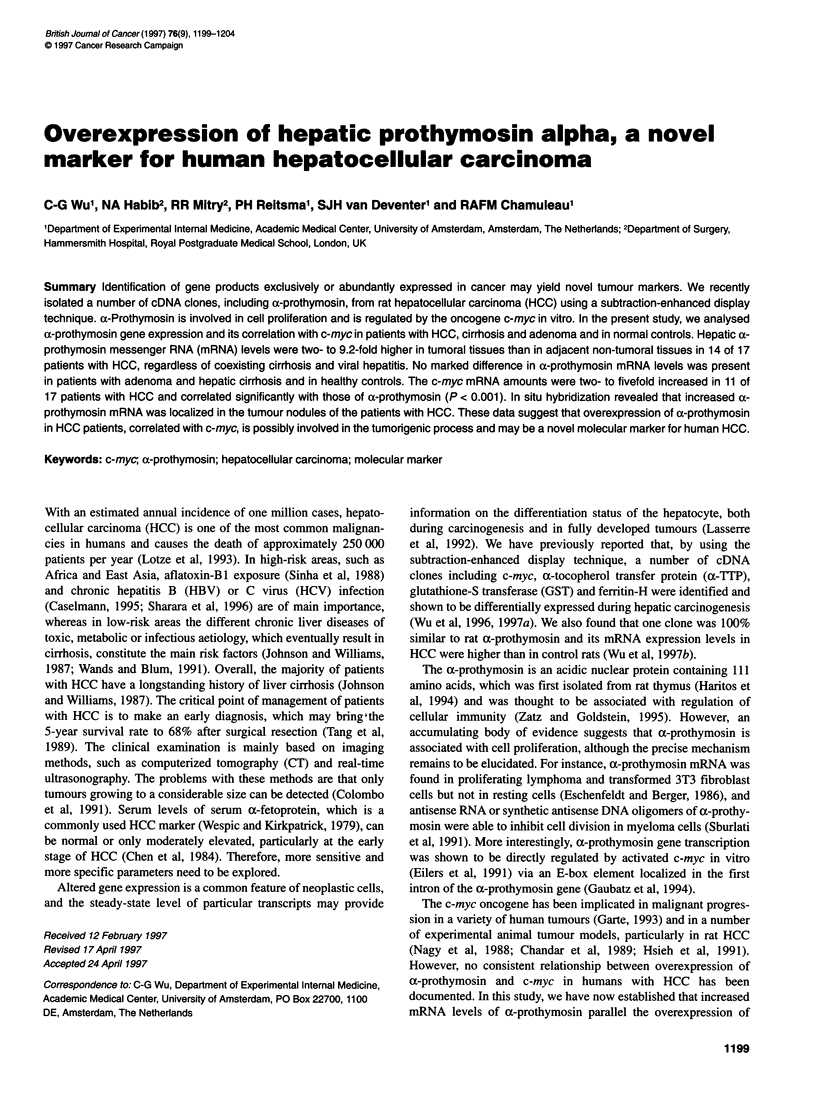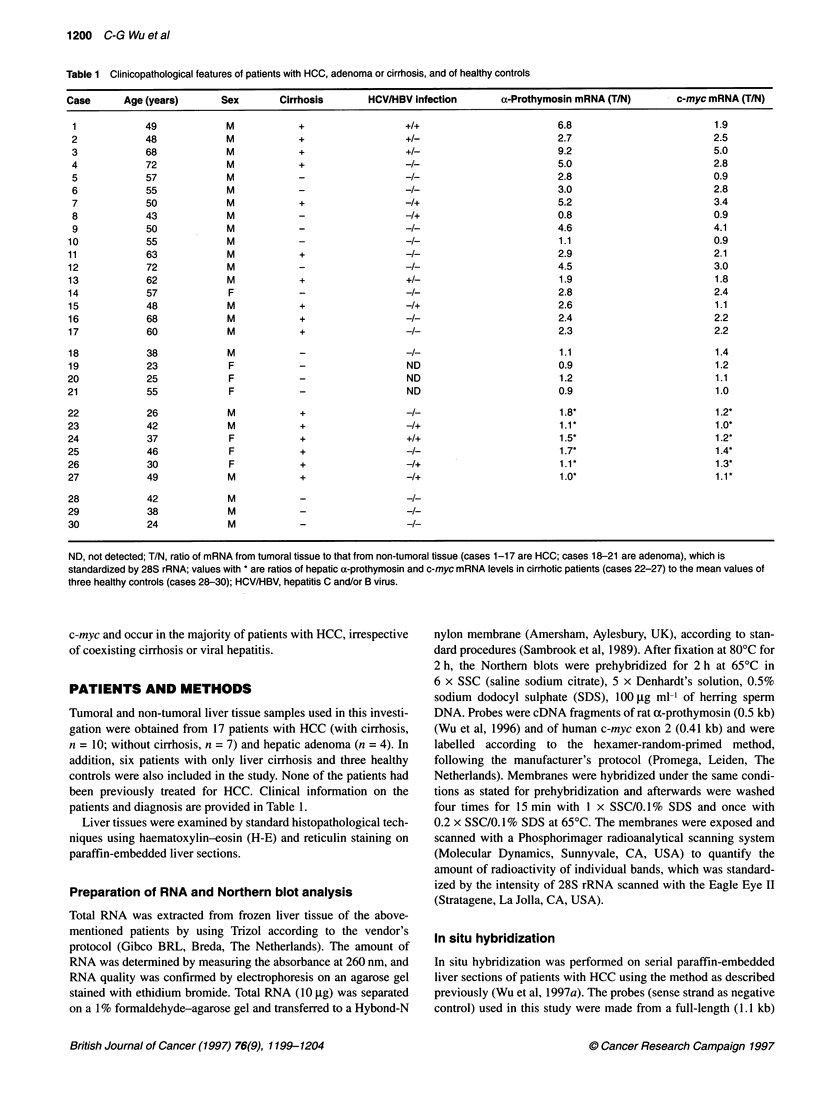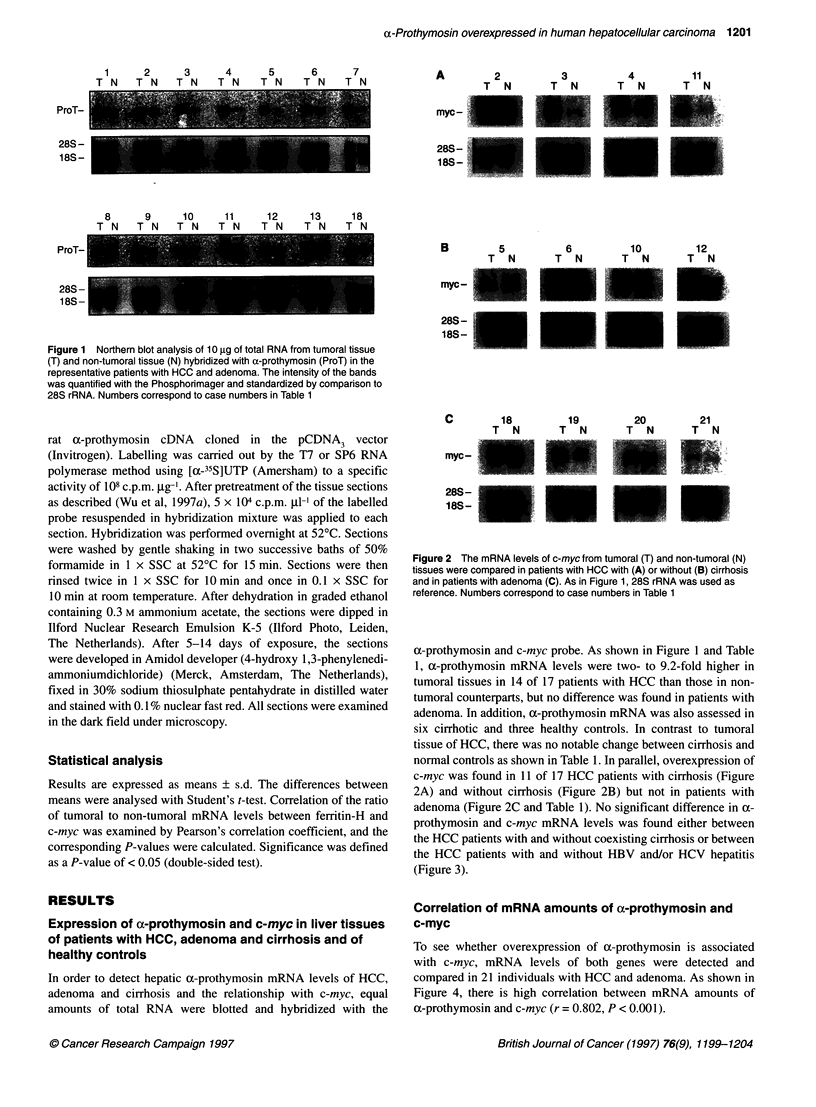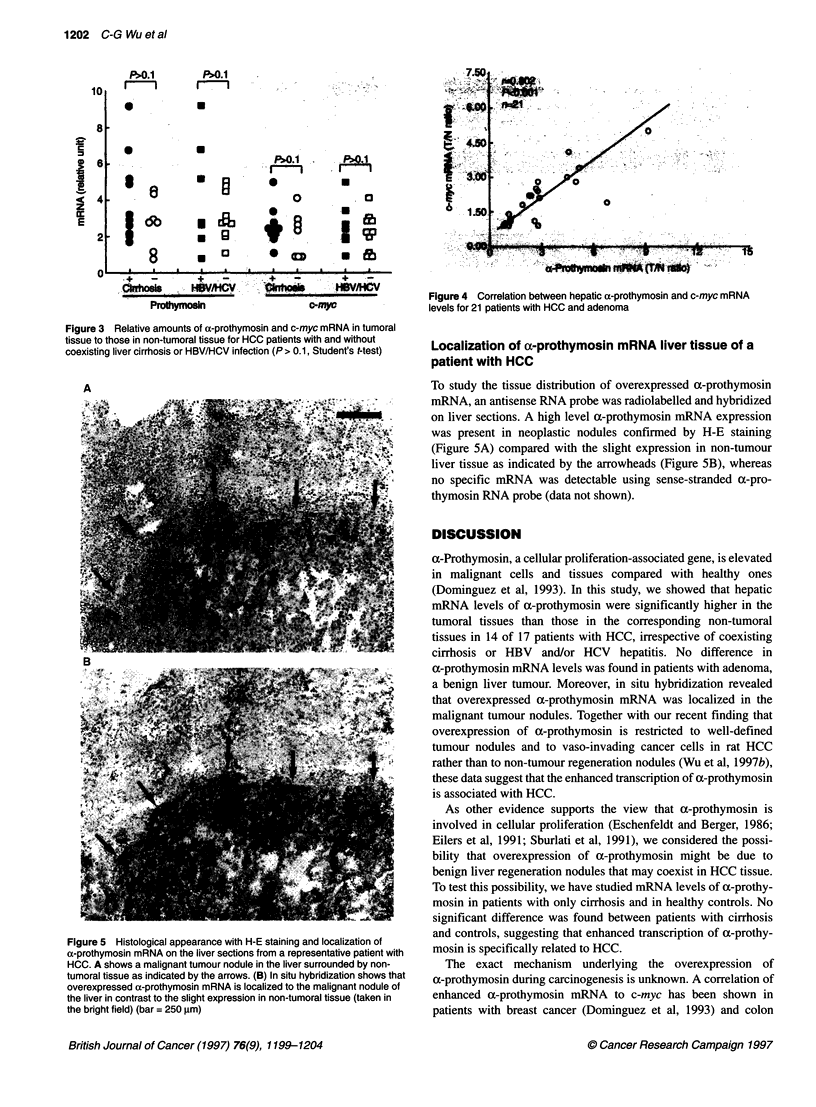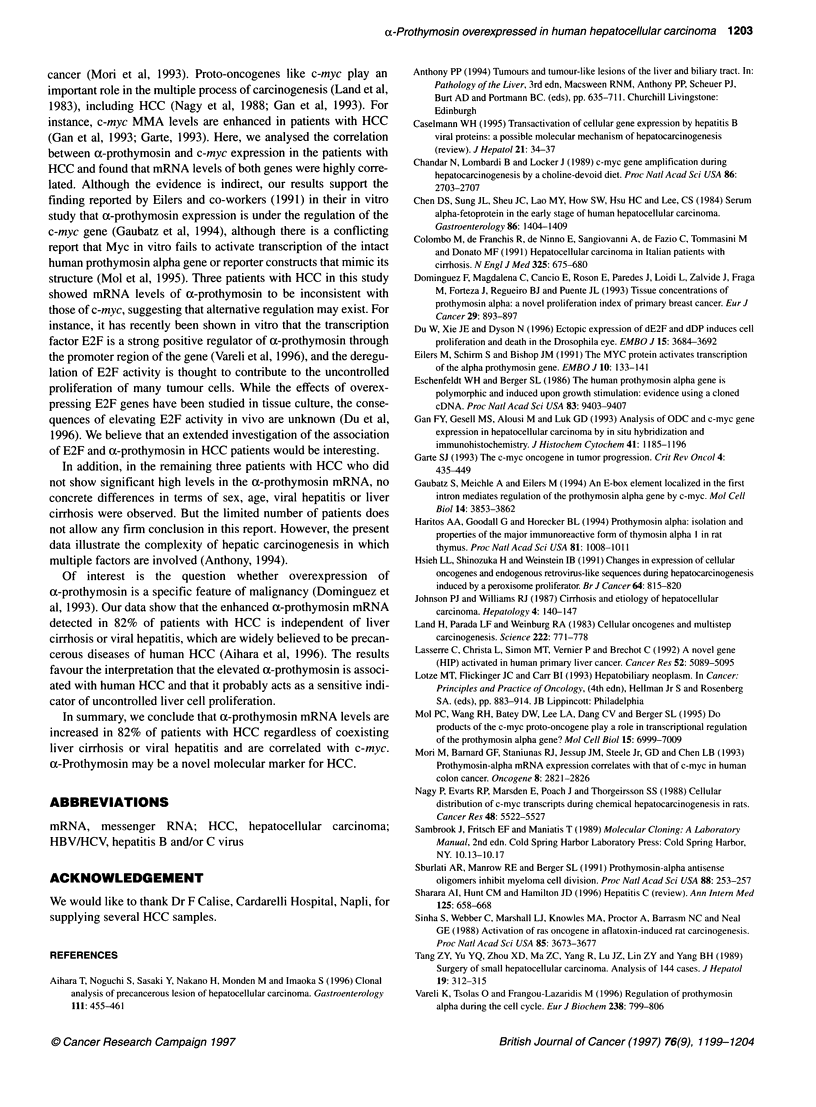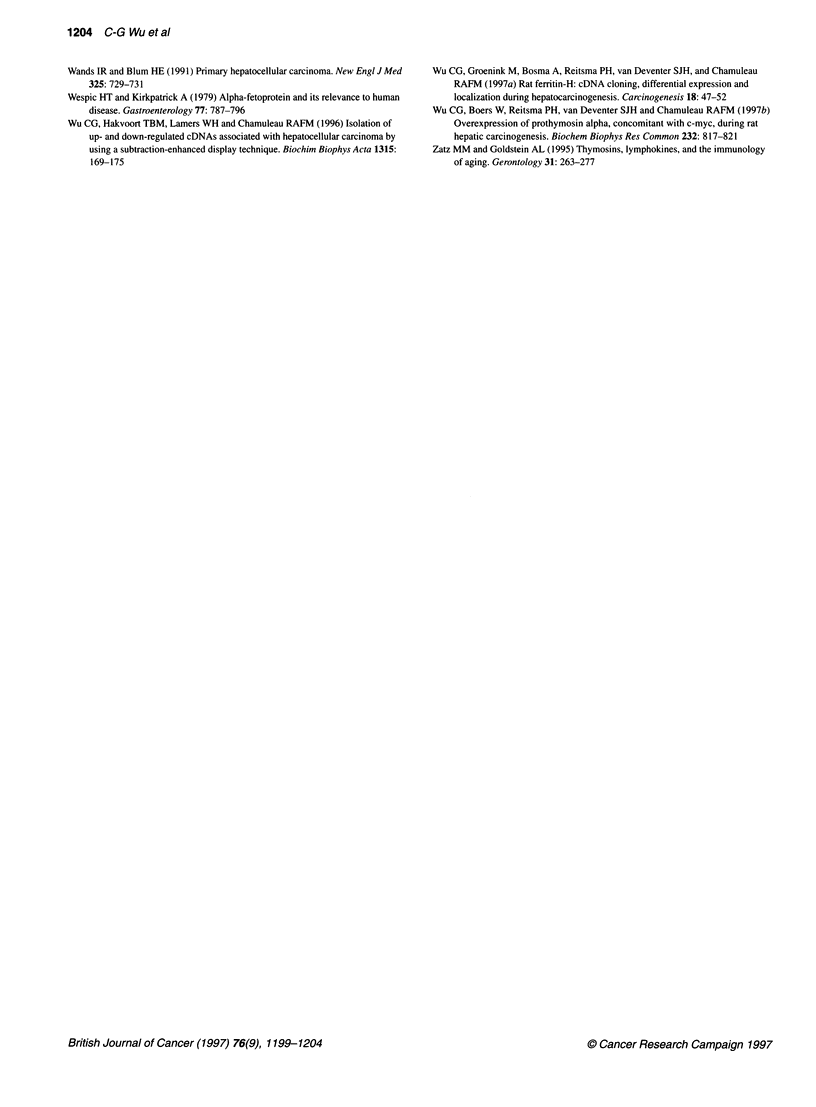# Overexpression of hepatic prothymosin alpha, a novel marker for human hepatocellular carcinoma.

**DOI:** 10.1038/bjc.1997.533

**Published:** 1997

**Authors:** C. G. Wu, N. A. Habib, R. R. Mitry, P. H. Reitsma, S. J. van Deventer, R. A. Chamuleau

**Affiliations:** Department of Experimental Internal Medicine, Academic Medical Center, University of Amsterdam, The Netherlands.

## Abstract

**Images:**


*British Journal of Cancer* (1997) **76**(9), 1199–1204
© 1997 Cancer Research Campaign

### Overexpression of hepatic prothymosin alpha, a novel marker for human hepatocellular carcinoma

**C-G Wu[1], NA Habib[2], RR Mitry[2], PH Reitsma[1], SJH van Deventer[1] and RAFM Chamuleau[1]**

[1]Department of Experimental Internal Medicine, Academic Medical Center, University of Amsterdam, Amsterdam, The Netherlands; [2]Department of Surgery, Hammersmith Hospital, Royal Postgraduate Medical School, London, UK

**Summary** Identification of gene products exclusively or abundantly expressed in cancer may yield novel tumour markers. We recently isolated a number of cDNA clones, including α-prothymosin, from rat hepatocellular carcinoma (HCC) using a subtraction-enhanced display technique. α-Prothymosin is involved in cell proliferation and is regulated by the oncogene c-*myc* in vitro. In the present study, we analysed α-prothymosin gene expression and its correlation with c-*myc* in patients with HCC, cirrhosis and adenoma and in normal controls. Hepatic α-prothymosin messenger RNA (mRNA) levels were two- to 9.2-fold higher in tumoral tissues than in adjacent non-tumoral tissues in 14 of 17 patients with HCC, regardless of coexisting cirrhosis and viral hepatitis. No marked difference in α-prothymosin mRNA levels was present in patients with adenoma and hepatic cirrhosis and in healthy controls. The c-*myc* mRNA amounts were two- to fivefold increased in 11 of 17 patients with HCC and correlated significantly with those of α-prothymosin ($P < 0.001$). In situ hybridization revealed that increased α-prothymosin mRNA was localized in the tumour nodules of the patients with HCC. These data suggest that overexpression of α-prothymosin in HCC patients, correlated with c-*myc*, is possibly involved in the tumorigenic process and may be a novel molecular marker for human HCC.

**Keywords:** c-*myc*; α-prothymosin; hepatocellular carcinoma; molecular marker

With an estimated annual incidence of one million cases, hepatocellular carcinoma (HCC) is one of the most common malignancies in humans and causes the death of approximately 250 000 patients per year (Lotze et al, 1993). In high-risk areas, such as Africa and East Asia, aflatoxin-B1 exposure (Sinha et al, 1988) and chronic hepatitis B (HBV) or C virus (HCV) infection (Caselmann, 1995; Sharara et al, 1996) are of main importance, whereas in low-risk areas the different chronic liver diseases of toxic, metabolic or infectious aetiology, which eventually result in cirrhosis, constitute the main risk factors (Johnson and Williams, 1987; Wands and Blum, 1991). Overall, the majority of patients with HCC have a longstanding history of liver cirrhosis (Johnson and Williams, 1987). The critical point of management of patients with HCC is to make an early diagnosis, which may bring the 5-year survival rate to 68% after surgical resection (Tang et al, 1989). The clinical examination is mainly based on imaging methods, such as computerized tomography (CT) and real-time ultrasonography. The problems with these methods are that only tumours growing to a considerable size can be detected (Colombo et al, 1991). Serum levels of serum α-fetoprotein, which is a commonly used HCC marker (Wespic and Kirkpatrick, 1979), can be normal or only moderately elevated, particularly at the early stage of HCC (Chen et al, 1984). Therefore, more sensitive and more specific parameters need to be explored.

Altered gene expression is a common feature of neoplastic cells, and the steady-state level of particular transcripts may provide information on the differentiation status of the hepatocyte, both during carcinogenesis and in fully developed tumours (Lasserre et al, 1992). We have previously reported that, by using the subtraction-enhanced display technique, a number of cDNA clones including c-*myc*, α-tocopherol transfer protein (α-TTP), glutathione-S transferase (GST) and ferritin-H were identified and shown to be differentially expressed during hepatic carcinogenesis (Wu et al, 1996, 1997*a*). We also found that one clone was 100% similar to rat α-prothymosin and its mRNA expression levels in HCC were higher than in control rats (Wu et al, 1997*b*).

The α-prothymosin is an acidic nuclear protein containing 111 amino acids, which was first isolated from rat thymus (Haritos et al, 1994) and was thought to be associated with regulation of cellular immunity (Zatz and Goldstein, 1995). However, an accumulating body of evidence suggests that α-prothymosin is associated with cell proliferation, although the precise mechanism remains to be elucidated. For instance, α-prothymosin mRNA was found in proliferating lymphoma and transformed 3T3 fibroblast cells but not in resting cells (Eschenfeldt and Berger, 1986), and antisense RNA or synthetic antisense DNA oligomers of α-prothymosin were able to inhibit cell division in myeloma cells (Sburlati et al, 1991). More interestingly, α-prothymosin gene transcription was shown to be directly regulated by activated c-*myc* in vitro (Eilers et al, 1991) via an E-box element localized in the first intron of the α-prothymosin gene (Gaubatz et al, 1994).

The c-*myc* oncogene has been implicated in malignant progression in a variety of human tumours (Garte, 1993) and in a number of experimental animal tumour models, particularly in rat HCC (Nagy et al, 1988; Chandar et al, 1989; Hsieh et al, 1991). However, no consistent relationship between overexpression of α-prothymosin and c-*myc* in humans with HCC has been documented. In this study, we have now established that increased mRNA levels of α-prothymosin parallel the overexpression of

*Received 12 February 1997*
*Revised 17 April 1997*
*Accepted 24 April 1997*

*Correspondence to:* C-G Wu, Department of Experimental Internal Medicine, Academic Medical Center, University of Amsterdam, PO Box 22700, 1100 DE, Amsterdam, The Netherlands

**Table 1** Clinicopathological features of patients with HCC, adenoma or cirrhosis, and of healthy controls

| Case | Age (years) | Sex | Cirrhosis | HCV/HBV infection | α-Prothymosin mRNA (T/N) | c-*myc* mRNA (T/N) |
|---|---|---|---|---|---|---|
| 1 | 49 | M | + | +/+ | 6.8 | 1.9 |
| 2 | 48 | M | + | +/– | 2.7 | 2.5 |
| 3 | 68 | M | + | +/– | 9.2 | 5.0 |
| 4 | 72 | M | + | –/– | 5.0 | 2.8 |
| 5 | 57 | M | – | –/– | 2.8 | 0.9 |
| 6 | 55 | M | – | –/– | 3.0 | 2.8 |
| 7 | 50 | M | + | –/+ | 5.2 | 3.4 |
| 8 | 43 | M | – | –/+ | 0.8 | 0.9 |
| 9 | 50 | M | – | –/– | 4.6 | 4.1 |
| 10 | 55 | M | – | –/– | 1.1 | 0.9 |
| 11 | 63 | M | + | –/– | 2.9 | 2.1 |
| 12 | 72 | M | – | –/– | 4.5 | 3.0 |
| 13 | 62 | M | + | +/– | 1.9 | 1.8 |
| 14 | 57 | F | – | –/– | 2.8 | 2.4 |
| 15 | 48 | M | + | –/+ | 2.6 | 1.1 |
| 16 | 68 | M | + | –/– | 2.4 | 2.2 |
| 17 | 60 | M | + | –/– | 2.3 | 2.2 |
| 18 | 38 | M | – | –/– | 1.1 | 1.4 |
| 19 | 23 | F | – | ND | 0.9 | 1.2 |
| 20 | 25 | F | – | ND | 1.2 | 1.1 |
| 21 | 55 | F | – | ND | 0.9 | 1.0 |
| 22 | 26 | M | + | –/– | 1.8* | 1.2* |
| 23 | 42 | M | + | –/+ | 1.1* | 1.0* |
| 24 | 37 | F | + | +/+ | 1.5* | 1.2* |
| 25 | 46 | F | + | –/– | 1.7* | 1.4* |
| 26 | 30 | F | + | –/+ | 1.1* | 1.3* |
| 27 | 49 | M | + | –/+ | 1.0* | 1.1* |
| 28 | 42 | M | – | –/– | | |
| 29 | 38 | M | – | –/– | | |
| 30 | 24 | M | – | –/– | | |

ND, not detected; T/N, ratio of mRNA from tumoral tissue to that from non-tumoral tissue (cases 1–17 are HCC; cases 18–21 are adenoma), which is standardized by 28S rRNA; values with * are ratios of hepatic α-prothymosin and c-*myc* mRNA levels in cirrhotic patients (cases 22–27) to the mean values of three healthy controls (cases 28–30); HCV/HBV, hepatitis C and/or B virus.

c-*myc* and occur in the majority of patients with HCC, irrespective of coexisting cirrhosis or viral hepatitis.

### PATIENTS AND METHODS

Tumoral and non-tumoral liver tissue samples used in this investigation were obtained from 17 patients with HCC (with cirrhosis, $n = 10$; without cirrhosis, $n = 7$) and hepatic adenoma ($n = 4$). In addition, six patients with only liver cirrhosis and three healthy controls were also included in the study. None of the patients had been previously treated for HCC. Clinical information on the patients and diagnosis are provided in Table 1.

Liver tissues were examined by standard histopathological techniques using haematoxylin–eosin (H-E) and reticulin staining on paraffin-embedded liver sections.

### Preparation of RNA and Northern blot analysis

Total RNA was extracted from frozen liver tissue of the above-mentioned patients by using Trizol according to the vendor's protocol (Gibco BRL, Breda, The Netherlands). The amount of RNA was determined by measuring the absorbance at 260 nm, and RNA quality was confirmed by electrophoresis on an agarose gel stained with ethidium bromide. Total RNA (10 μg) was separated on a 1% formaldehyde–agarose gel and transferred to a Hybond-N nylon membrane (Amersham, Aylesbury, UK), according to standard procedures (Sambrook et al, 1989). After fixation at 80°C for 2 h, the Northern blots were prehybridized for 2 h at 65°C in 6 × SSC (saline sodium citrate), 5 × Denhardt's solution, 0.5% sodium dodocyl sulphate (SDS), 100 μg $ml^{-1}$ of herring sperm DNA. Probes were cDNA fragments of rat α-prothymosin (0.5 kb) (Wu et al, 1996) and of human c-*myc* exon 2 (0.41 kb) and were labelled according to the hexamer-random-primed method, following the manufacturer's protocol (Promega, Leiden, The Netherlands). Membranes were hybridized under the same conditions as stated for prehybridization and afterwards were washed four times for 15 min with 1 × SSC/0.1% SDS and once with 0.2 × SSC/0.1% SDS at 65°C. The membranes were exposed and scanned with a Phosphorimager radioanalytical scanning system (Molecular Dynamics, Sunnyvale, CA, USA) to quantify the amount of radioactivity of individual bands, which was standardized by the intensity of 28S rRNA scanned with the Eagle Eye II (Stratagene, La Jolla, CA, USA).

### In situ hybridization

In situ hybridization was performed on serial paraffin-embedded liver sections of patients with HCC using the method as described previously (Wu et al, 1997*a*). The probes (sense strand as negative control) used in this study were made from a full-length (1.1 kb)

© Cancer Research Campaign 1997

**Figure 1** Northern blot analysis of 10 μg of total RNA from tumoral tissue (T) and non-tumoral tissue (N) hybridized with α-prothymosin (ProT) in the representative patients with HCC and adenoma. The intensity of the bands was quantified with the Phosphorimager and standardized by comparison to 28S rRNA. Numbers correspond to case numbers in Table 1

rat α-prothymosin cDNA cloned in the pCDNA$_3$ vector (Invitrogen). Labelling was carried out by the T7 or SP6 RNA polymerase method using [α-$^{35}$S]UTP (Amersham) to a specific activity of $10^8$ c.p.m. $\mu g^{-1}$. After pretreatment of the tissue sections as described (Wu et al, 1997*a*), $5 \times 10^4$ c.p.m. $\mu l^{-1}$ of the labelled probe resuspended in hybridization mixture was applied to each section. Hybridization was performed overnight at 52°C. Sections were washed by gentle shaking in two successive baths of 50% formamide in 1 × SSC at 52°C for 15 min. Sections were then rinsed twice in 1 × SSC for 10 min and once in 0.1 × SSC for 10 min at room temperature. After dehydration in graded ethanol containing 0.3 M ammonium acetate, the sections were dipped in Ilford Nuclear Research Emulsion K-5 (Ilford Photo, Leiden, The Netherlands). After 5–14 days of exposure, the sections were developed in Amidol developer (4-hydroxy 1,3-phenylenediammoniumdichloride) (Merck, Amsterdam, The Netherlands), fixed in 30% sodium thiosulphate pentahydrate in distilled water and stained with 0.1% nuclear fast red. All sections were examined in the dark field under microscopy.

### Statistical analysis

Results are expressed as means ± s.d. The differences between means were analysed with Student's *t*-test. Correlation of the ratio of tumoral to non-tumoral mRNA levels between ferritin-H and c-*myc* was examined by Pearson's correlation coefficient, and the corresponding *P*-values were calculated. Significance was defined as a *P*-value of < 0.05 (double-sided test).

### RESULTS

### Expression of α-prothymosin and c-*myc* in liver tissues of patients with HCC, adenoma and cirrhosis and of healthy controls

In order to detect hepatic α-prothymosin mRNA levels of HCC, adenoma and cirrhosis and the relationship with c-*myc*, equal amounts of total RNA were blotted and hybridized with the α-prothymosin and c-*myc* probe. As shown in Figure 1 and Table 1, α-prothymosin mRNA levels were two- to 9.2-fold higher in tumoral tissues in 14 of 17 patients with HCC than those in non-tumoral counterparts, but no difference was found in patients with adenoma. In addition, α-prothymosin mRNA was also assessed in six cirrhotic and three healthy controls. In contrast to tumoral tissue of HCC, there was no notable change between cirrhosis and normal controls as shown in Table 1. In parallel, overexpression of c-*myc* was found in 11 of 17 HCC patients with cirrhosis (Figure 2A) and without cirrhosis (Figure 2B) but not in patients with adenoma (Figure 2C and Table 1). No significant difference in α-prothymosin and c-*myc* mRNA levels was found either between the HCC patients with and without coexisting cirrhosis or between the HCC patients with and without HBV and/or HCV hepatitis (Figure 3).

**Figure 2** The mRNA levels of c-*myc* from tumoral (T) and non-tumoral (N) tissues were compared in patients with HCC with (**A**) or without (**B**) cirrhosis and in patients with adenoma (**C**). As in Figure 1, 28S rRNA was used as reference. Numbers correspond to case numbers in Table 1

### Correlation of mRNA amounts of α-prothymosin and c-myc

To see whether overexpression of α-prothymosin is associated with c-*myc*, mRNA levels of both genes were detected and compared in 21 individuals with HCC and adenoma. As shown in Figure 4, there is high correlation between mRNA amounts of α-prothymosin and c-*myc* ($r = 0.802$, $P < 0.001$).

© Cancer Research Campaign 1997

**Figure 3** Relative amounts of α-prothymosin and c-*myc* mRNA in tumoral tissue to those in non-tumoral tissue for HCC patients with and without coexisting liver cirrhosis or HBV/HCV infection ($P > 0.1$, Student's *t*-test)

**Figure 5** Histological appearance with H-E staining and localization of α-prothymosin mRNA on the liver sections from a representative patient with HCC. **A** shows a malignant tumour nodule in the liver surrounded by non-tumoral tissue as indicated by the arrows. (**B**) In situ hybridization shows that overexpressed α-prothymosin mRNA is localized to the malignant nodule of the liver in contrast to the slight expression in non-tumoral tissue (taken in the bright field) (bar = 250 μm)

**Figure 4** Correlation between hepatic α-prothymosin and c-*myc* mRNA levels for 21 patients with HCC and adenoma

### Localization of α-prothymosin mRNA liver tissue of a patient with HCC

To study the tissue distribution of overexpressed α-prothymosin mRNA, an antisense RNA probe was radiolabelled and hybridized on liver sections. A high level α-prothymosin mRNA expression was present in neoplastic nodules confirmed by H-E staining (Figure 5A) compared with the slight expression in non-tumour liver tissue as indicated by the arrowheads (Figure 5B), whereas no specific mRNA was detectable using sense-stranded α-prothymosin RNA probe (data not shown).

### DISCUSSION

α-Prothymosin, a cellular proliferation-associated gene, is elevated in malignant cells and tissues compared with healthy ones (Dominguez et al, 1993). In this study, we showed that hepatic mRNA levels of α-prothymosin were significantly higher in the tumoral tissues than those in the corresponding non-tumoral tissues in 14 of 17 patients with HCC, irrespective of coexisting cirrhosis or HBV and/or HCV hepatitis. No difference in α-prothymosin mRNA levels was found in patients with adenoma, a benign liver tumour. Moreover, in situ hybridization revealed that overexpressed α-prothymosin mRNA was localized in the malignant tumour nodules. Together with our recent finding that overexpression of α-prothymosin is restricted to well-defined tumour nodules and to vaso-invading cancer cells in rat HCC rather than to non-tumour regeneration nodules (Wu et al, 1997*b*), these data suggest that the enhanced transcription of α-prothymosin is associated with HCC.

As other evidence supports the view that α-prothymosin is involved in cellular proliferation (Eschenfeldt and Berger, 1986; Eilers et al, 1991; Sburlati et al, 1991), we considered the possibility that overexpression of α-prothymosin might be due to benign liver regeneration nodules that may coexist in HCC tissue. To test this possibility, we have studied mRNA levels of α-prothymosin in patients with only cirrhosis and in healthy controls. No significant difference was found between patients with cirrhosis and controls, suggesting that enhanced transcription of α-prothymosin is specifically related to HCC.

The exact mechanism underlying the overexpression of α-prothymosin during carcinogenesis is unknown. A correlation of enhanced α-prothymosin mRNA to c-*myc* has been shown in patients with breast cancer (Dominguez et al, 1993) and colon

© Cancer Research Campaign 1997

cancer (Mori et al, 1993). Proto-oncogenes like c-*myc* play an important role in the multiple process of carcinogenesis (Land et al, 1983), including HCC (Nagy et al, 1988; Gan et al, 1993). For instance, c-*myc* MMA levels are enhanced in patients with HCC (Gan et al, 1993; Garte, 1993). Here, we analysed the correlation between α-prothymosin and c-*myc* expression in the patients with HCC and found that mRNA levels of both genes were highly correlated. Although the evidence is indirect, our results support the finding reported by Eilers and co-workers (1991) in their in vitro study that α-prothymosin expression is under the regulation of the c-*myc* gene (Gaubatz et al, 1994), although there is a conflicting report that Myc in vitro fails to activate transcription of the intact human prothymosin alpha gene or reporter constructs that mimic its structure (Mol et al, 1995). Three patients with HCC in this study showed mRNA levels of α-prothymosin to be inconsistent with those of c-*myc*, suggesting that alternative regulation may exist. For instance, it has recently been shown in vitro that the transcription factor E2F is a strong positive regulator of α-prothymosin through the promoter region of the gene (Vareli et al, 1996), and the deregulation of E2F activity is thought to contribute to the uncontrolled proliferation of many tumour cells. While the effects of overexpressing E2F genes have been studied in tissue culture, the consequences of elevating E2F activity in vivo are unknown (Du et al, 1996). We believe that an extended investigation of the association of E2F and α-prothymosin in HCC patients would be interesting.

In addition, in the remaining three patients with HCC who did not show significant high levels in the α-prothymosin mRNA, no concrete differences in terms of sex, age, viral hepatitis or liver cirrhosis were observed. But the limited number of patients does not allow any firm conclusion in this report. However, the present data illustrate the complexity of hepatic carcinogenesis in which multiple factors are involved (Anthony, 1994).

Of interest is the question whether overexpression of α-prothymosin is a specific feature of malignancy (Dominguez et al, 1993). Our data show that the enhanced α-prothymosin mRNA detected in 82% of patients with HCC is independent of liver cirrhosis or viral hepatitis, which are widely believed to be precancerous diseases of human HCC (Aihara et al, 1996). The results favour the interpretation that the elevated α-prothymosin is associated with human HCC and that it probably acts as a sensitive indicator of uncontrolled liver cell proliferation.

In summary, we conclude that α-prothymosin mRNA levels are increased in 82% of patients with HCC regardless of coexisting liver cirrhosis or viral hepatitis and are correlated with c-*myc*. α-Prothymosin may be a novel molecular marker for HCC.

### ABBREVIATIONS

mRNA, messenger RNA; HCC, hepatocellular carcinoma; HBV/HCV, hepatitis B and/or C virus

### ACKNOWLEDGEMENT

We would like to thank Dr F Calise, Cardarelli Hospital, Napli, for supplying several HCC samples.

### REFERENCES

Aihara T, Noguchi S, Sasaki Y, Nakano H, Monden M and Imaoka S (1996) Clonal analysis of precancerous lesion of hepatocellular carcinoma. *Gastroenterology* **111**: 455–461

Anthony PP (1994) Tumours and tumour-like lesions of the liver and biliary tract. In: *Pathology of the Liver*, 3rd edn, Macsween RNM, Anthony PP, Scheuer PJ, Burt AD and Portmann BC. (eds), pp. 635–711. Churchill Livingstone: Edinburgh

Caselmann WH (1995) Transactivation of cellular gene expression by hepatitis B viral proteins: a possible molecular mechanism of hepatocarcinogenesis (review). *J Hepatol* **21**: 34–37

Chandar N, Lombardi B and Locker J (1989) c-myc gene amplification during hepatocarcinogenesis by a choline-devoid diet. *Proc Natl Acad Sci USA* **86**: 2703–2707

Chen DS, Sung JL, Sheu JC, Lao MY, How SW, Hsu HC and Lee, CS (1984) Serum alpha-fetoprotein in the early stage of human hepatocellular carcinoma. *Gastroenterology* **86**: 1404–1409

Colombo M, de Franchis R, de Ninno E, Sangiovanni A, de Fazio C, Tommasini M and Donato MF (1991) Hepatocellular carcinoma in Italian patients with cirrhosis. *N Engl J Med* **325**: 675–680

Dominguez F, Magdalena C, Cancio E, Roson E, Paredes J, Loidi L, Zalvide J, Fraga M, Forteza J, Regueiro BJ and Puente JL (1993) Tissue concentrations of prothymosin alpha: a novel proliferation index of primary breast cancer. *Eur J Cancer* **29**: 893–897

Du W, Xie JE and Dyson N (1996) Ectopic expression of dE2F and dDP induces cell proliferation and death in the Drosophila eye. *EMBO J* **15**: 3684–3692

Eilers M, Schirm S and Bishop JM (1991) The MYC protein activates transcription of the alpha prothymosin gene. *EMBO J* **10**: 133–141

Eschenfeldt WH and Berger SL (1986) The human prothymosin alpha gene is polymorphic and induced upon growth stimulation: evidence using a cloned cDNA. *Proc Natl Acad Sci USA* **83**: 9403–9407

Gan FY, Gesell MS, Alousi M and Luk GD (1993) Analysis of ODC and c-myc gene expression in hepatocellular carcinoma by in situ hybridization and immunohistochemistry. *J Histochem Cytochem* **41**: 1185–1196

Garte SJ (1993) The c-myc oncogene in tumor progression. *Crit Rev Oncol* **4**: 435–449

Gaubatz S, Meichle A and Eilers M (1994) An E-box element localized in the first intron mediates regulation of the prothymosin alpha gene by c-myc. *Mol Cell Biol* **14**: 3853–3862

Haritos AA, Goodall G and Horecker BL (1994) Prothymosin alpha: isolation and properties of the major immunoreactive form of thymosin alpha 1 in rat thymus. *Proc Natl Acad Sci USA* **81**: 1008–1011

Hsieh LL, Shinozuka H and Weinstein IB (1991) Changes in expression of cellular oncogenes and endogenous retrovirus-like sequences during hepatocarcinogenesis induced by a peroxisome proliferator. *Br J Cancer* **64**: 815–820

Johnson PJ and Williams RJ (1987) Cirrhosis and etiology of hepatocellular carcinoma. *Hepatology* **4**: 140–147

Land H, Parada LF and Weinburg RA (1983) Cellular oncogenes and multistep carcinogenesis. *Science* **222**: 771–778

Lasserre C, Christa L, Simon MT, Vernier P and Brechot C (1992) A novel gene (HIP) activated in human primary liver cancer. *Cancer Res* **52**: 5089–5095

Lotze MT, Flickinger JC and Carr BI (1993) Hepatobiliary neoplasm. In *Cancer: Principles and Practice of Oncology*, (4th edn), Hellman Jr S and Rosenberg SA. (eds), pp. 883–914. JB Lippincott: Philadelphia

Mol PC, Wang RH, Batey DW, Lee LA, Dang CV and Berger SL (1995) Do products of the c-myc proto-oncogene play a role in transcriptional regulation of the prothymosin alpha gene? *Mol Cell Biol* **15**: 6999–7009

Mori M, Barnard GF, Staniunas RJ, Jessup JM, Steele Jr, GD and Chen LB (1993) Prothymosin-alpha mRNA expression correlates with that of c-myc in human colon cancer. *Oncogene* **8**: 2821–2826

Nagy P, Evarts RP, Marsden E, Poach J and Thorgeirsson SS (1988) Cellular distribution of c-myc transcripts during chemical hepatocarcinogenesis in rats. *Cancer Res* **48**: 5522–5527

Sambrook J, Fritsch EF and Maniatis T (1989) *Molecular Cloning: A Laboratory Manual*, 2nd edn. Cold Spring Harbor Laboratory Press: Cold Spring Harbor, NY. 10.13–10.17

Sburlati AR, Manrow RE and Berger SL (1991) Prothymosin-alpha antisense oligomers inhibit myeloma cell division. *Proc Natl Acad Sci USA* **88**: 253–257

Sharara AI, Hunt CM and Hamilton JD (1996) Hepatitis C (review). *Ann Intern Med* **125**: 658–668

Sinha S, Webber C, Marshall LJ, Knowles MA, Proctor A, Barrasm NC and Neal GE (1988) Activation of ras oncogene in aflatoxin-induced rat carcinogenesis. *Proc Natl Acad Sci USA* **85**: 3673–3677

Tang ZY, Yu YQ, Zhou XD, Ma ZC, Yang R, Lu JZ, Lin ZY and Yang BH (1989) Surgery of small hepatocellular carcinoma. Analysis of 144 cases. *J Hepatol* **19**: 312–315

Vareli K, Tsolas O and Frangou-Lazaridis M (1996) Regulation of prothymosin alpha during the cell cycle. *Eur J Biochem* **238**: 799–806

© Cancer Research Campaign 1997

Wands IR and Blum HE (1991) Primary hepatocellular carcinoma. *New Engl J Med* **325**: 729–731

Wespic HT and Kirkpatrick A (1979) Alpha-fetoprotein and its relevance to human disease. *Gastroenterology* **77**: 787–796

Wu CG, Hakvoort TBM, Lamers WH and Chamuleau RAFM (1996) Isolation of up- and down-regulated cDNAs associated with hepatocellular carcinoma by using a subtraction-enhanced display technique. *Biochim Biophys Acta* **1315**: 169–175

Wu CG, Groenink M, Bosma A, Reitsma PH, van Deventer SJH, and Chamuleau RAFM (1997*a*) Rat ferritin-H: cDNA cloning, differential expression and localization during hepatocarcinogenesis. *Carcinogenesis* **18**: 47–52

Wu CG, Boers W, Reitsma PH, van Deventer SJH and Chamuleau RAFM (1997*b*) Overexpression of prothymosin alpha, concomitant with c-myc, during rat hepatic carcinogenesis. *Biochem Biophys Res Common* **232**: 817–821

Zatz MM and Goldstein AL (1995) Thymosins, lymphokines, and the immunology of aging. *Gerontology* **31**: 263–277

*© Cancer Research Campaign 1997*